# Murine IL-2 outperforms human IL-2 in supporting the survival of naïve mouse CD8 T cells in vitro

**DOI:** 10.26508/lsa.202603711

**Published:** 2026-09-24

**Authors:** Yangle Yu, Tete I Obot, Madaleine M Niznikiewicz, Craig M Podszus, Julio S Dos-Santos, Elena L Pobezinskaya, Priyadharshini Devarajan, Leonid A Pobezinsky, Brian S Sheridan

**Affiliations:** 1 Department of Microbiology and Immunology, Renaissance School of Medicine, Stony Brook University , Stony Brook, NY, USA; 2 Center for Infectious Diseases, Renaissance School of Medicine, Stony Brook University , Stony Brook, NY, USA; 3 Department of Veterinary and Animal Sciences, University of Massachusetts , Amherst, MA, USA

## Abstract

Much of the immunology community relies on human IL-2 to support murine CD8 T-cell expansion in vitro. Although human and murine IL-2 share high similarity in amino acid sequence and exhibit similar biological activity, the potential cross-species implications of this practice remain largely unexamined, representing a significant yet overlooked variable in many in vitro studies that serve as the foundation of T-cell biology. Here, we compared the effects of human and murine IL-2 on CD8 T cells in vitro to uncover potential differences in T-cell biology. Murine IL-2 more effectively supported the survival of mouse naïve CD8 T cells in vitro and induced a distinct phenotype of IL-2 receptor expression on activated mouse CD8 T cells. In addition, murine IL-2 promoted enhanced antigen-independent expansion of memory-like CD8 T cells from naïve animals. Collectively, these findings should encourage laboratories to reevaluate their human IL-2 dosing strategies or consider transitioning to murine IL-2 for in vitro studies using murine CD8 T cells.

## Abstract (precis)

Murine IL-2 promotes the survival of naïve mouse CD8 T cells in vitro compared with human IL-2 and induces a distinct expression pattern of IL-2 receptors in activated CD8 T cells.

## Introduction

Interleukin 2 (IL-2) is a critical cytokine involved in regulating CD8 T-cell activation and is essential for supporting the expansion of activated CD8 T cells in vitro and in vivo (Obar et al, 2010; Spolski et al, 2018). Although IL-2 is primarily produced by activated CD4 T cells, other cells including CD8 T cells, γδ T cells, NK cells, and a range of innate immune cells can also produce IL-2 to varying degrees (Whyte et al, 2022; Shouse et al, 2024). IL-2 receptors (IL-2Rs) are composed of three subunits, IL-2Rα (CD25), IL-2Rβ (CD122), and IL-2Rγ (CD132; γC). IL-2Rβ and IL-2Rγ form the intermediate-affinity receptor (K_d_ ∼10^−9^ M), whereas incorporation of IL-2Rα generates the high-affinity receptor complex (K_d_ ∼10^−11^ M) (Liao et al, 2013). Both intermediate- and high-affinity IL-2Rs are biologically functional; IL-2 initially binds IL-2Rα, inducing a conformational change of IL-2Rα that increases the affinity for the IL-2Rβ and IL-2Rγ heterodimer for IL-2, the subunits responsible for initiating intracellular signaling in T cells (Nelson et al, 1994; Spolski et al, 2018).

IL-2 is highly conserved across species. Human and murine IL-2 share ∼63% amino acid sequence identity, including the signal peptide, implying broadly similar biological activity (Kashima et al, 1985). Human IL-2 promotes the proliferation of both human and murine T cells, whereas murine IL-2 was less effective at stimulating human T cells (Mosmann et al, 1987). This asymmetry is attributed to species-specific differences in IL-2R binding. Human IL-2 exhibits an ∼100-fold higher affinity for the human high-affinity IL-2R compared with murine IL-2 (Collins, 1989). In contrast, human and murine IL-2 bind the murine high-affinity IL-2R with a comparable affinity consistent with the similar biological activity on murine T-cell proliferation (Collins, 1989; Liu et al, 1996). These data suggest that human and murine IL-2 exhibit species-specific differences in CD8 T-cell biology. Despite this, human IL-2 is widely used in culturing murine CD8 T cells in vitro (Opferman et al, 1999; Klebanoff et al, 2004; Mueller et al, 2008; Wonderlich et al, 2018; Huang et al., 2019, 2021; Macchi et al, 2023; Wu et al, 2023; Tan et al, 2024). However, it remains unclear whether the species-specific differences in IL-2 influence its role in the survival and activation of naïve CD8 T cells in vitro. In this study, we examined the functional differences between human and murine IL-2 during in vitro mouse CD8 T-cell cultures. Regardless of source, IL-2 exerted a strong dose-dependent effect on the survival of mouse naïve CD8 T cells in vitro. However, at concentrations routinely used by most laboratories, murine IL-2 was substantially more effective at supporting naïve CD8 T-cell survival. In addition, both human and murine IL-2 enhanced the sensitivity of CD8 T cells to peptide stimulation, yet each cytokine induced a distinct profile of CD122 and CD25 expression. Altogether, our findings highlight important species-specific functional differences in IL-2 during in vitro CD8 T-cell culture.

## Results

### Murine IL-2 improves the survival of naïve CD8 T cells in vitro

**Figure S1.**
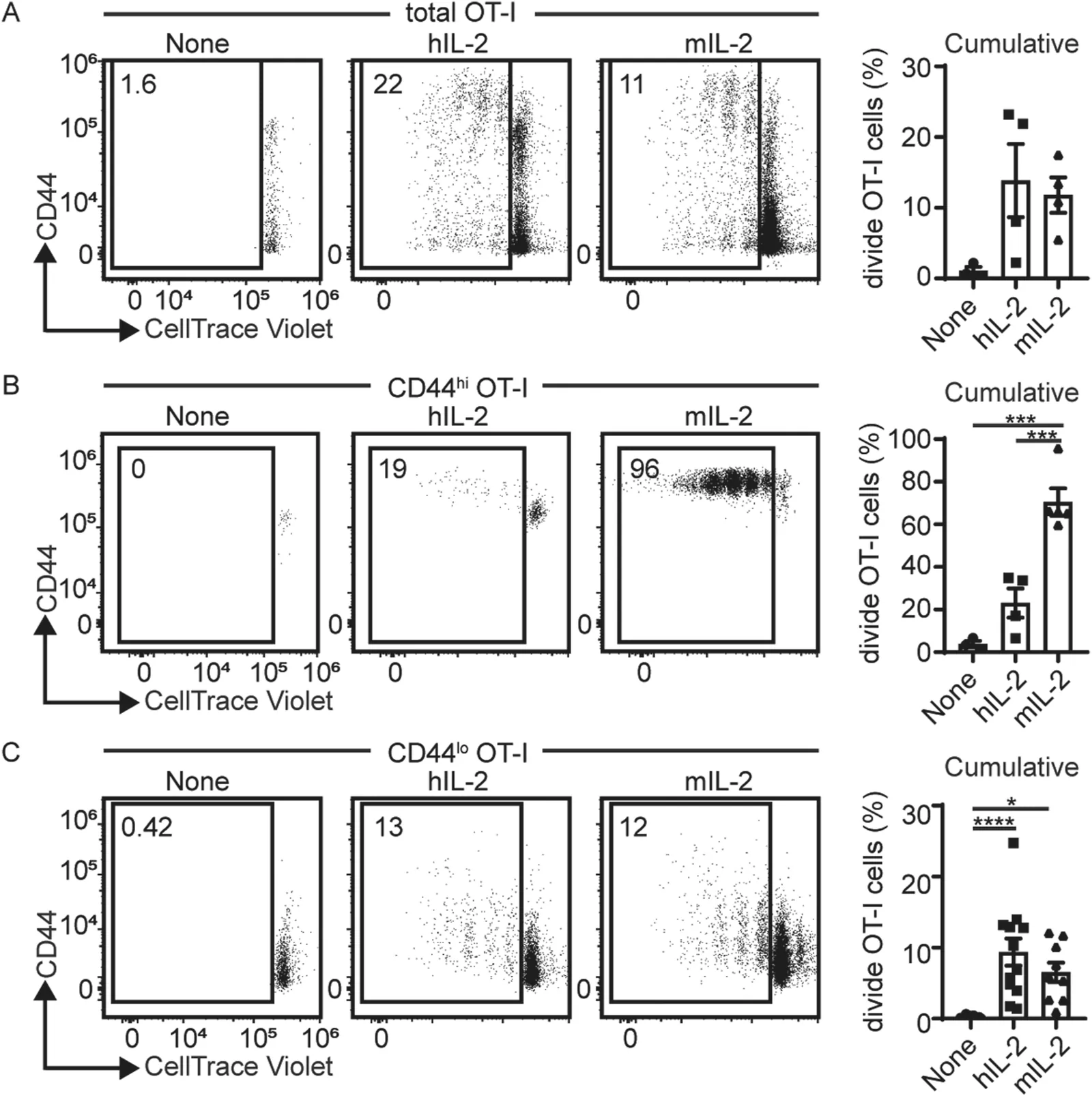
IL-2 promotes the proliferation of CD44^hi^ OT-I cells in vitro. 1 × 10^5^ CTV-labeled OT-I cells from naïve mice were cultured with or without 200 U/ml hIL-2 or mIL-2 for 3 d. Representative dot plots are shown for total OT-I cells (A), CD44^hi^ OT-I cells (B), and CD44^lo^ OT-I cells (C). The means from technical triplicates are pooled from four or more independent experiments. Each symbol depicts the mean of each condition in each independent experiment. The data are expressed as the mean ± SEM. One-way ANOVA with a post hoc *t* test was performed. *, *P* ≤ 0.05; ***, *P* ≤ 0.001; ****, *P* ≤ 0.0001. mIL-2, murine IL-2; hIL-2, human IL-2.

The impact of human and murine IL-2 was assessed on OT-I cells isolated from naïve OT-I TCR transgenic mice. OT-I cells were cultured for 3 d in the presence or absence of 200 U/ml of human or murine IL-2 in the absence of TCR stimulation. OT-I cells modestly proliferated in response to human or murine IL-2 without TCR engagement in vitro (Fig S1A). IL-2 is critical for supporting CD8 T-cell proliferation after activation and is sufficient to drive effector T-cell proliferation in vitro in the absence of antigenic stimulation (Manjunath et al, 2001; Obar et al, 2010). Consistent with this, most proliferating cells appeared CD44^hi^ from the initial division (Fig S1A). Therefore, we sought to determine whether IL-2–induced expansion in the absence of antigenic stimulation was primarily driven by preexisting memory phenotype CD8 T cells. Memory phenotype CD44^hi^ and CD44^lo^ OT-I cells were sort-purified from naïve OT-I mice and cultured for 3 d in the presence or absence of 200 U/ml of human or murine IL-2 to independently assess IL-2 responsiveness. 50–200 U/ml of IL-2 appears to be the most commonly used concentration of human IL-2 in the culture of CD8 T cells (Lee et al, 2014; Yu et al, 2016; Mandal et al, 2023). Memory phenotype CD44^hi^ OT-I cells displayed greater proliferation in response to IL-2 regardless of the source. However, murine IL-2 induced substantially higher levels of proliferation compared with human IL-2 (Fig S1B). In contrast, antigen-inexperienced CD44^lo^ OT-I cells from naïve OT-I mice underwent minimal proliferation in response to human or murine IL-2, and dividing cells induced by IL-2 maintained lower expression levels of CD44 (Fig S1C). Thus, IL-2 preferentially drives the expansion of memory phenotype CD44^hi^ CD8 T cells in the absence of antigenic stimulation and this effect is more pronounced in response to murine IL-2.

**Figure 1.**
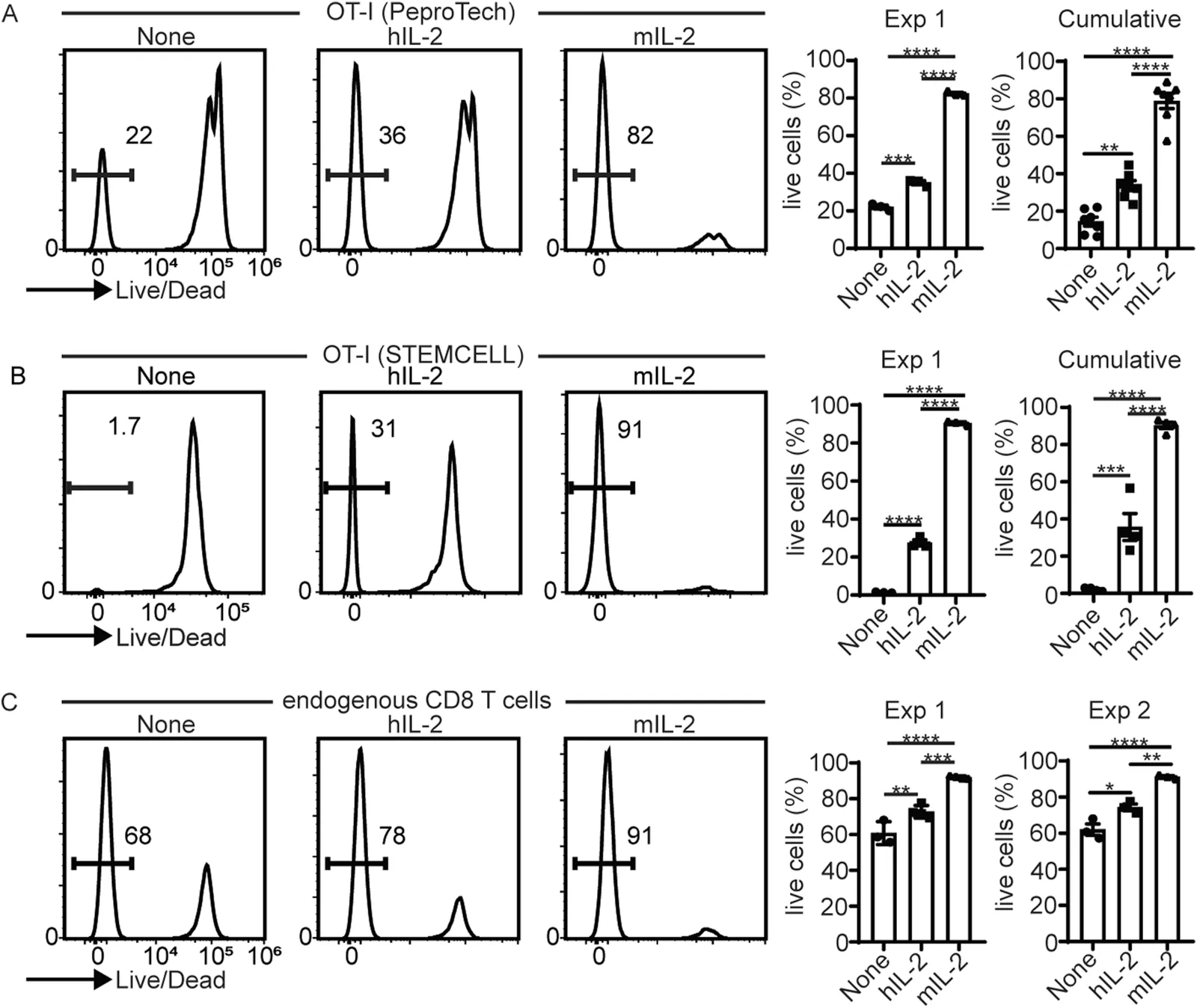
mIL-2 improves the survival of naïve CD8 T cells in vitro. 1 × 10^5^ CD44^lo^ CD8 T cells were isolated from the spleens of naïve mice by MACS purification and cultured with or without 200 U/ml of hIL-2 or mIL-2 for 3 d. The viability of CD44^lo^ CD8 T cells was analyzed by a viability dye. **(A, B)** Representative histograms showing viability of CD44^lo^ OT-I cells cultured with IL-2 from PeproTech (A) or STEMCELL (B). The data in bar graphs (Exp 1) are representative of an independent experiment with technical triplicates. Bar graphs (cumulative) are pooled from four or more independent experiments. Each symbol depicts the mean of each condition from independent experiments. **(C)** Representative histograms showing viability of endogenous CD44^lo^ CD8 T cells cultured with IL-2 from PeproTech. The bar graphs depict two independent experiments with technical triplicates. The data are expressed as the mean ± SEM. One-way ANOVA with a post hoc *t* test was performed. *, *P* ≤ 0.05; **, *P* ≤ 0.01; ***, *P* ≤ 0.001; ****, *P* ≤ 0.0001. mIL-2, murine IL-2; hIL-2, human IL-2.

**Figure S2.**
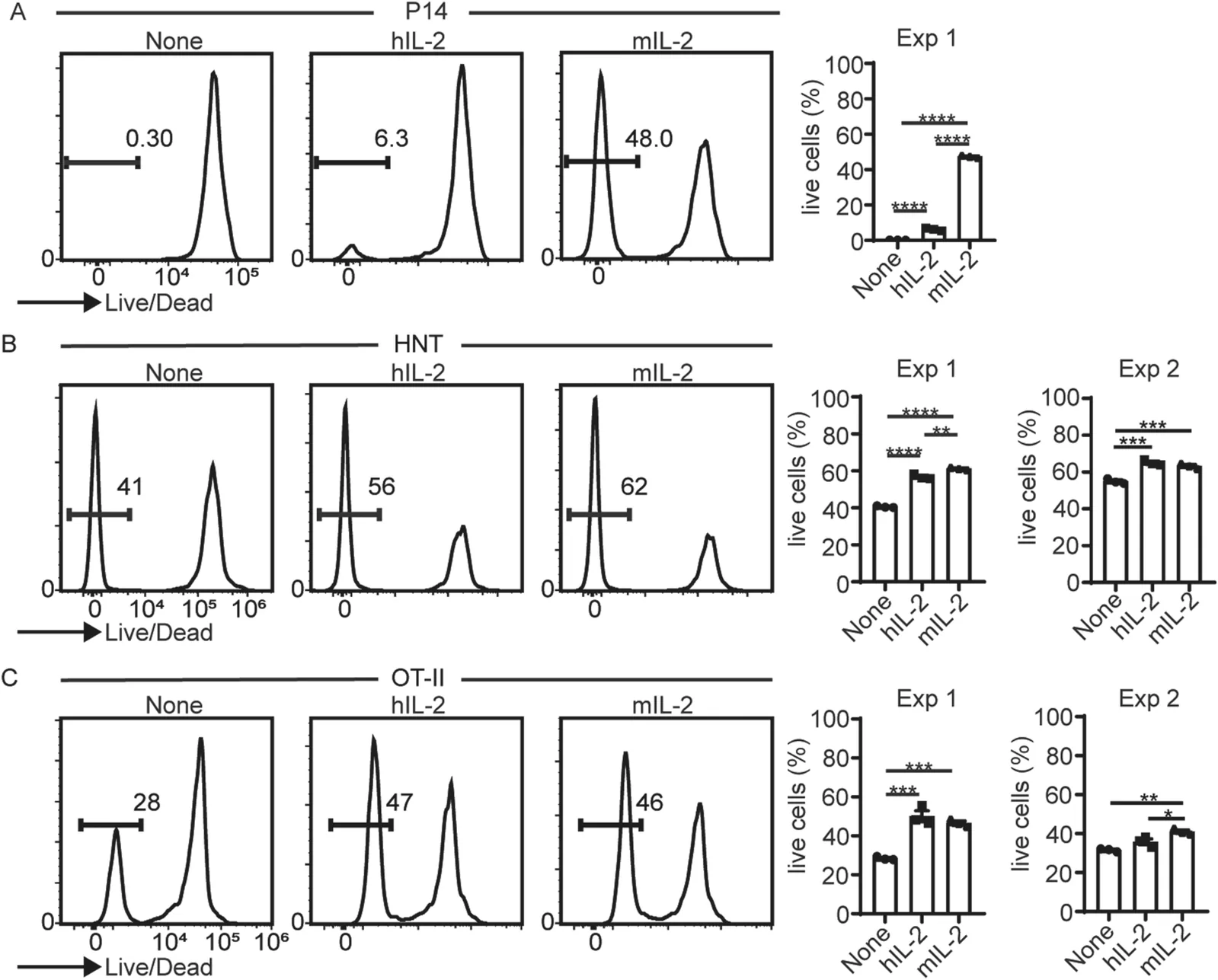
IL-2 improves the survival of naïve CD4 T cells in vitro. 1 × 10^5^ CD44^lo^ P14 cells or total splenocytes from HNT or OT-II mice were cultured with or without 200 U/ml hIL-2 or mIL-2 for 3 d. The viability of naïve P14 (A), HNT (B), or OT-II (C) is shown as representative histograms and depicted as the mean ± SEM. The graphs depict independent experiments with technical triplicates for each condition. One-way ANOVA with a post hoc *t* test was performed. *, *P* ≤ 0.05; **, *P* ≤ 0.01; ***, *P* ≤ 0.001; ****, *P* ≤ 0.0001. mIL-2, murine IL-2; hIL-2, human IL-2.

We next sought to determine whether human and murine IL-2 differ in their ability to support naïve CD8 T cells in vitro. We isolated CD44^lo^ OT-I cells from the spleens of naïve OT-I mice and cultured them with either human or murine IL-2 for 3 d. Both human and murine IL-2 enhanced the viability of naïve OT-I cells in vitro, but murine IL-2 demonstrated substantially improved viability compared with human IL-2 (Fig 1A). A similar effect was observed with IL-2 from a distinct vendor (Fig 1B). To further validate the protective effect of murine IL-2 in CD8 T cells, we purified CD44^lo^ CD8 T cells from naïve C57BL/6 mice and P14 TCR transgenic mice with CD8 T cells specific to the lymphocytic choriomeningitis virus gp33 epitope to determine their responsiveness to human and murine IL-2. Consistent with the results from CD44^lo^ OT-I CD8 T cells, murine IL-2 was superior to human IL-2 in promoting survival of CD44^lo^ P14 cells and endogenous polyclonal CD44^lo^ CD8 T cells in vitro (Figs 1C and S2A).

IL-2 is also essential for the survival, proliferation, and function of regulatory T cells (Davidson et al, 2007; Zheng et al, 2007; Liao et al, 2013). Loss of IL-2 signaling hampers the survival of regulatory T cells in the periphery (Fan et al, 2018). To assess whether murine IL-2 exerts a similar effect on CD4 T cells in vitro, we cultured CD44^lo^ HNT cells (specific to the influenza virus hemagglutinin epitope) or OT-II cells (specific to ovalbumin epitope) from naïve transgenic mice with either human or murine IL-2 for 3 d. Both human and murine IL-2 similarly enhanced the viability of CD44^lo^ CD4 T cells in vitro (Fig S2B and C). However, murine IL-2 did not provide a consistent or substantial benefit on the survival of CD44^lo^ CD4 T cells compared with human IL-2. Thus, the benefit of murine IL-2 over human IL-2 is primarily restricted to CD8 T cells.

### The impact of IL-2 on survival is dose-dependent

**Figure 2.**
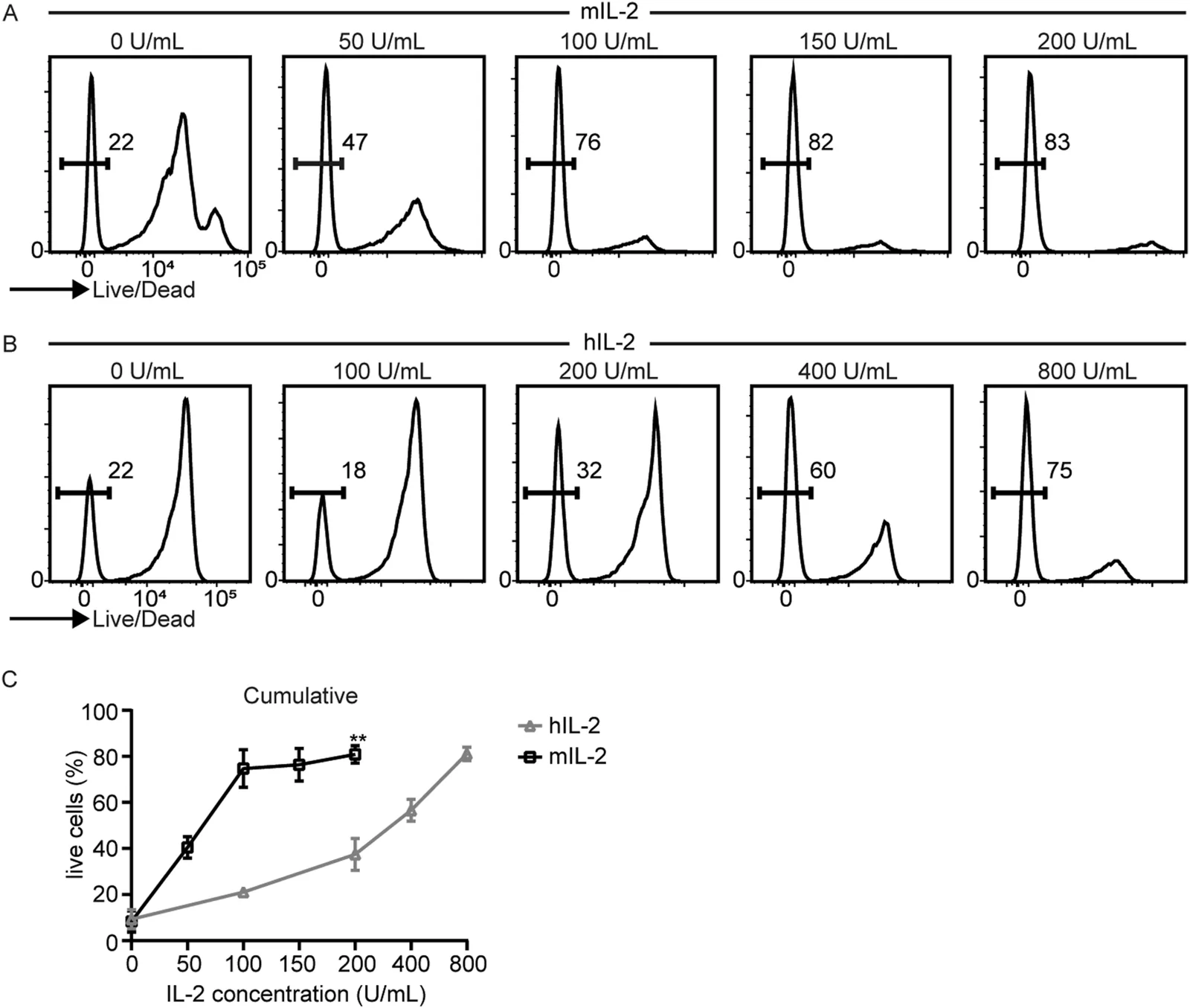
Impact of IL-2 on survival is dose-dependent. 1 × 10^5^ CD44^lo^ OT-I cells were isolated from spleens and cultured with the indicated concentrations of IL-2 for 3 d. The viability of CD44^lo^ CD8 T cells was analyzed by a viability dye. Representative histograms show the viability of CD44^lo^ OT-I cells cultured with mIL-2 (A) or hIL-2 (B). **(C)** Means from technical triplicates are pooled from four independent experiments (two for PeproTech and two for STEMCELL) except for 100 U/ml hIL-2. The data are expressed as the mean ± SEM, n = 4. An unpaired *t* test was performed for 200 U/ml IL-2. **, *P* ≤ 0.01. mIL-2, murine IL-2; hIL-2, human IL-2.

To investigate whether the dose of IL-2 impacts the survival of naïve CD8 T cells in vitro, we cultured CD44^lo^ OT-I cells with varying concentrations of murine IL-2 for 3 d. Increasing doses of murine IL-2 progressively enhanced the viability of CD44^lo^ OT-I cells, with 150–200 U/ml murine IL-2 providing the strongest protective effect (Fig 2A). Likewise, the viability of CD44^lo^ OT-I cells was augmented by an increasing dose of human IL-2 (Fig 2B). However, an approximately four- to eightfold higher concentration of human IL-2 was required to achieve viability levels comparable to those induced by murine IL-2 (Fig 2C).

High concentrations of IL-2 are sufficient to induce the activation and proliferation of naïve CD8 T cells, accompanied by the up-regulation of CD44 and CD25, which is associated with antigen-independent activation of the PI3K/AKT pathway (Cho et al, 2013). Both murine and human IL-2 promoted the proliferation of a small population of CD44^lo^ OT-I cells in vitro (Fig S1C). These suggest that the protective effect of IL-2 on naïve CD8 T-cell survival is dose-dependent and murine IL-2 confers effective survival support at substantially lower concentrations than human IL-2.

### IL-2 enhances the sensitivity of CD8 T cells to antigenic stimulation

**Figure 3.**
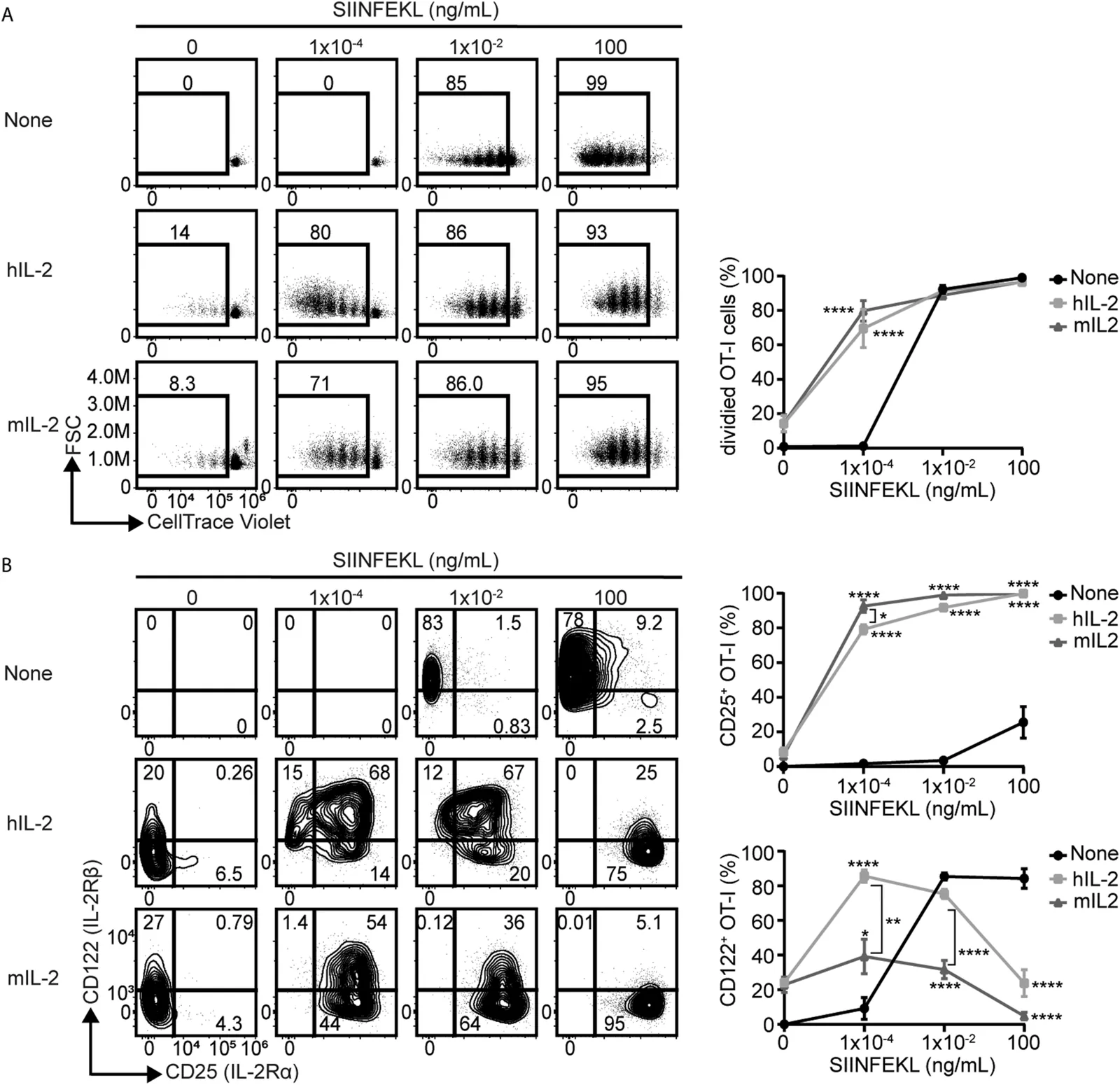
IL-2 enhances the sensitivity of CD8 T cells to antigenic stimulation. 1 × 10^5^ CTV-labeled CD44^lo^ OT-I cells were cultured with the indicated concentrations of SIINFEKL peptide in the presence of 200 U/ml hIL-2 or mIL-2 for 3 d. **(A)** Representative histograms displaying CTV dilution of total OT-I cells are shown. **(B)** Representative contour plots displaying the expression of CD25 and CD122 on CTV-diluted OT-I cells are shown. The means from technical triplicates are pooled from four independent experiments (two for PeproTech and two for STEMCELL). The frequency of divided OT-I cells among total OT-I cells, the frequency of CD25^+^ OT-I cells among divided OT-I cells, and the frequency of CD122^+^ OT-I cells among divided OT-I cells are shown as the mean ± SEM, n = 4. One-way ANOVA with a post hoc *t* test was performed. *, *P* ≤ 0.05; **, *P* ≤ 0.01; ****, *P* ≤ 0.0001. mIL-2, murine IL-2; hIL-2, human IL-2.

**Figure S3.**
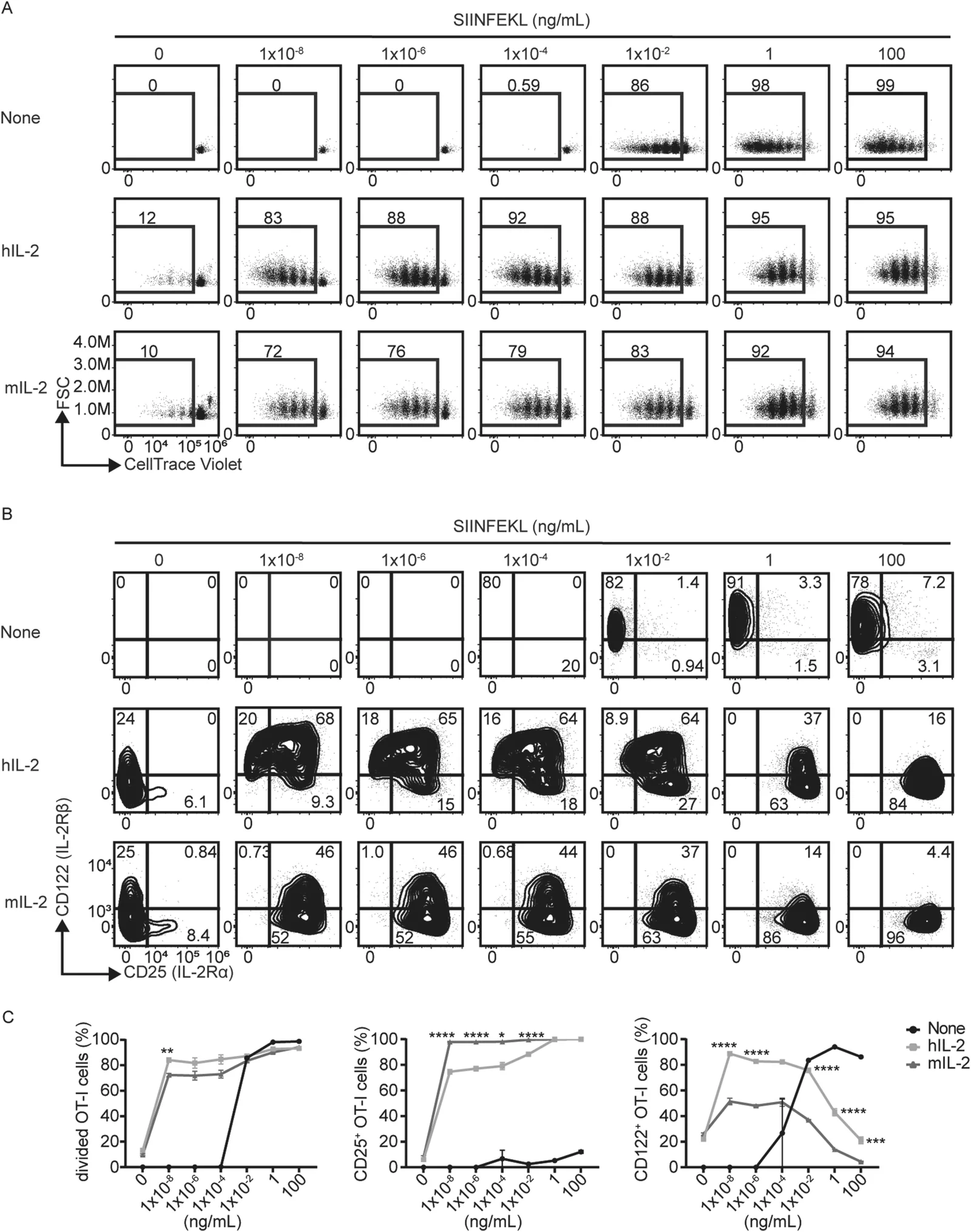
IL-2 enhances the sensitivity of CD8 T cells to antigenic stimulation. 1 × 10^5^ CTV-labeled CD44^lo^ OT-I cells were cultured with the indicated concentrations of SIINFEKL peptide in the presence of 200 U/ml hIL-2 or mIL-2 for 3 d. These data included a wider range of peptide concentrations than Fig 3. **(A)** Representative dot plots displaying CTV dilution of total OT-I cells are shown. **(B)** Representative contour plots displaying the expression of CD25 and CD122 on CTV-diluted OT-I cells are shown. Data are representative of two independent experiments (with PeproTech IL-2) with technical triplicates from one experiment shown. **(C)** Frequency of divided OT-I cells among total OT-I cells, the frequency of CD25^+^ OT-I cells among divided OT-I cells, and the frequency of CD122^+^ OT-I cells among divided OT-I cells are shown as the mean ± SEM. One-way ANOVA with a post hoc *t* test was performed on mIL-2 versus hIL-2. *, *P* ≤ 0.05; **, *P* ≤ 0.01; ***, *P* ≤ 0.001; ****, *P* ≤ 0.0001. mIL-2, murine IL-2; hIL-2, human IL-2.

To determine the role of IL-2 in the CD8 T-cell response, we activated CD44^lo^ OT-I cells with OVA_257-264_ peptide (SIINFEKL) in the presence of either human or murine IL-2. CD44^lo^ OT-I cells were labeled with CellTrace Violet (CTV) before stimulation, and the CTV dilution was analyzed on day 3. The concentration of SIINFEKL and other CD8 T-cell epitopes that are commonly used to stimulate CD8 T cells in vitro is in the 0.1–1 μg/ml range (Kamimura & Bevan, 2007; Maroof et al, 2009; Hale et al, 2024). Within a concentration range of 0.01–100 ng/ml, CD44^lo^ OT-I cells efficiently proliferated regardless of the presence or absence of IL-2. However, in the absence of IL-2, SIINFEKL concentrations below 0.01 ng/ml failed to induce the proliferation of CD44^lo^ OT-I cells in vitro. Both human and murine IL-2 comparably enhanced the sensitivity of CD44^lo^ OT-I cells to SIINFEKL (Figs 3A and S3A and C).

The expression of IL-2Rs on effector CD8 T cells is tightly regulated by TCR engagement and IL-2 signaling. The expression of CD25 on activated CD8 T cells in vivo is transient, reflecting the brief burst of IL-2 production after infection (Kalia et al, 2010). In addition, blockade of IL-2 signaling suppresses CD25 induction during the early phase of activation (Kalia et al, 2010), and IL-2–driven STAT5 activation is required for the up-regulation of CD25 (Kim et al, 2001). Consistently, CD25 was poorly up-regulated on dividing OT-I cells in vitro in the absence of IL-2. Both human and murine IL-2 promoted CD25 expression on activated OT-I cells, with murine IL-2 showing a stronger effect at lower SIINFEKL concentrations (Figs 3B and S3B and C). Most activated OT-I cells up-regulated CD122 without IL-2 treatment. However, fewer OT-I cells expressed CD122 to appreciable levels in response to murine IL-2. Notably, fewer OT-I cells exposed to murine or human IL-2 expressed CD122 as the concentration of SIINFEKL peptide increased. Altogether, these results suggest that the administration of IL-2 enhances the sensitivity of CD8 T cells to antigen in vitro and murine and human IL-2 differentially shape the IL-2R expression profile of CD8 T cells in an antigen dose-dependent manner.

## Discussion

IL-2 is a widely used T-cell growth factor that supports the expansion of CD8 T cells in vitro. Human and murine IL-2 share high similarity in amino acid sequence including the signal peptide (Kashima et al, 1985). Meanwhile, the amino acid sequence of IL-2Rs between human and mouse shares 60% homology, especially in the transmembrane and cytoplasmic regions that are highly conserved (Shimuzu et al, 1985). However, little is known about species-specific differences in IL-2 with respect to the survival and activation of murine CD8 T cells in vitro. Here, we demonstrate three key differences between murine and human IL-2 on murine CD8 T cells in vitro: (1) murine IL-2 provides enhanced support for the survival of naïve CD8 T cells in vitro, (2) murine and human IL-2 lead to distinct IL-2R expression patterns after stimulation, and (3) murine IL-2 promotes enhanced antigen-independent division of CD44^hi^ CD8 T cells from naïve mice.

The high-affinity IL-2R is composed of three subunits including IL-2Rα, IL-2Rβ, and IL-2Rγ. Even though there is substantial homology between human and murine IL-2 and their receptor chains, IL-2Rα has been shown to predominantly determine species-specific ligand-binding preferences (Liu et al, 1996). Human IL-2Rα fails to bind murine IL-2 effectively, resulting in weak interactions between murine IL-2 and the human IL-2R heterotrimers (Liu et al, 1996). Consistently, murine IL-2 is less effective at activating human T cells, whereas human IL-2 can stimulate the proliferation of both human and mouse T cells at similar concentrations (Mosmann et al, 1987). In contrast, murine IL-2R heterotrimers can bind both murine and human IL-2 effectively (Liu et al, 1996). However, IL-2Rα is barely expressed on naïve CD8 T cells, whereas IL-2Rβ and IL-2Rγ are constitutively expressed on resting T cells (Kim et al, 2001; Malek, 2008; Kalia et al, 2010; Spolski et al, 2018). Thus, species-specific differences may occur on naïve T cells. Indeed, our findings indicate that murine IL-2 outperforms human IL-2 in enhancing the survival of murine naïve CD8 T cells in vitro. Human IL-2 requires substantially higher concentrations to achieve viability levels of CD44^lo^ OT-I cells comparable to those induced by murine IL-2. IL-2 can also bind the IL-2Rβ and IL-2Rγ heterodimer with intermediate affinity (Wang & Smith, 1987). Our data demonstrate that there are species-specific differences in IL-2 responsiveness of naïve CD8 T cells and suggest that murine IL-2 has higher affinity for the IL-2Rβ and IL-2Rγ heterodimer. The binding of IL-2 has been reported to prevent mitochondrial damage by enhancing the expression of the anti-apoptotic Bcl-2 and IAP family members and sequestering the pro-apoptotic Bax and Bak highlighting a potential mechanism of IL-2–mediated survival of naïve CD8 T cells in vitro (Bhattacharya et al, 2005; Ross & Cantrell, 2018; Mustafa et al, 2024).

Both human and murine IL-2 can also enhance the survival of naïve CD4 T cells in vitro. However, the viability of CD44^lo^ CD4 T cells is lower than that of CD44^lo^ CD8 T cells at the same concentration of IL-2. Moreover, the magnitude of the difference between human and murine IL-2 is smaller than that observed in CD8 T cells. Neither naïve CD4 nor naïve CD8 T cells express appreciable levels of IL-2Rα. In addition, the expression of IL-2Rβ on naïve CD4 T cells appears limited to undetectable (Boyman & Sprent, 2012; Au-Yeung et al., 2017). In contrast, naïve CD8 T cells display a low but detectable level of IL-2Rβ expression (Zhang et al, 1998; Boyman & Sprent, 2012; Smith et al, 2017). The differential expression patterns of IL-2Rβ on naïve CD4 and CD8 T cells are consistent with our observations that CD4 T-cell viability in response to human and murine IL-2 is less substantial than that of CD8 T cells.

Previous studies demonstrated that human and murine IL-2 exerted similar activity in promoting mouse T-cell proliferation (Bleackley et al, 1985; Mosmann et al, 1987). Consistently, our data show that both human and murine IL-2 comparably promote naïve CD8 T-cell expansion to peptide stimulation with only minimal differences emerging at very low concentrations of peptide. Although both human and murine IL-2 enhanced CD25 expression, murine IL-2 promoted higher CD25 expression at a low SIINFEKL concentration. IL-2 is also essential for CD8 T-cell memory development. CD25^hi^ CD8 T cells after primary infection preferentially proliferate and differentiate into KLRG1^+^ short-lived effector T cells, whereas CD25^lo^ cells tend to differentiate into long-lived memory precursor effector T cells (Kalia et al, 2010). In addition, a higher proportion of OT-I cells express IL-2Rβ when culturing with human IL-2 than with murine IL-2 at the same concentration of SIINFEKL. Persistent expression of CD122 has been observed in CD8 T cells during chronic lymphocytic choriomeningitis virus and hepatitis C virus infections, where T cells exhibit functional exhaustion with increased PD-1 and Eomes (Beltra et al, 2016). Loss of IL-2Rβ on CD8 T cells restrains the expression of inhibitory receptors (PD-1, Lag3, 2B4, and Tim-3) and facilitates CD8 T cells to differentiate into CD127^hi^ KLRG-1^lo^ long-lived memory precursor effector T cells (Mathieu et al, 2015; Beltra et al, 2016). These indicate that the differential expression of CD25 and CD122 on CD8 T cells is closely associated with T-cell function, effector cell differentiation, and memory potential.

Altogether, our findings demonstrate that murine IL-2 markedly enhances the survival of naïve mouse CD8 T cells in vitro without inducing T-cell activation. Naïve CD8 T-cell culture is an important technique for studying T-cell priming and differentiation. Increased survival of naïve CD8 T cells facilitates prolonged interaction between T cells and APCs during culture, thereby enhancing the efficiency of antigen presentation of APCs in vitro. Finally, the use of murine IL-2 allows for more appropriate controls as it promotes the survival of a tangible population of unstimulated naïve CD8 T cells after in vitro culture. We also show that murine and human IL-2 can induce distinct changes in IL-2R expression patterns, which may alter the downstream biology of activated CD8 T cells. Given the widespread use of recombinant IL-2 in T-cell cultures, our data suggest that IL-2 derived from different species may influence the differentiation and function of CD8 T cells in vitro and warrant careful consideration of the most appropriate culturing conditions.

## Materials and Methods

### Mice

C57BL/6J and OT-II TCR transgenic mice were purchased from The Jackson Laboratory. OT-I Rag1^−/−^ mice expressing CD45.1 or CD45.2 were bred and maintained at Stony Brook University under specific-pathogen-free conditions. HNT. Thy1.2 mice were provided by Dr. Priyadharshini Devarajan (Stony Brook University). Splenocytes from P14 TCR transgenic mice were provided by Dr. Leonid A. Pobezinsky (University of Massachusetts, Amherst). For each experiment, 8- to 16-wk-old mice were used. All experimental animal procedures were carried out in accordance with National Institutes of Health (NIH) guidelines and approved by the Stony Brook University Institutional Animal Care and Use Committee.

### T-cell isolation

CD44^lo^ CD8 or CD4 T cells were purified from the spleens of naïve mice by magnetic separation. Briefly, spleens were mashed through 70-μm cell strainers (Falcon) to obtain single-cell suspensions. Splenocytes were then incubated with biotin-conjugated antibodies against CD44, CD19, CD11b, CD11c, CD49b, MHC-II, and either CD4 (for CD44^lo^ CD8 enrichment) or CD8α (for CD44^lo^ CD4 enrichment) followed by incubation with anti-biotin MicroBeads (Miltenyi Biotec) and enrichment on MACS Separator (Miltenyi Biotec). The purity of CD44^lo^ CD8 or CD4 T cells was >95%. CD44^hi^ OT-I cells were obtained by sort purification from OT-I spleens with a purity >95%.

### IL-2

Murine and human IL-2 were purchased from PeproTech (Cat# 212-12 and 200-02) and STEMCELL Technologies (Cat# 78081 and 78220). Both human and murine IL-2 are expressed by *Escherichia coli* and have ≥95% purity with endotoxin levels of <0.1 ng/μg. Cytokines were initially reconstituted following the manufacturer’s instruction and then diluted in PBS containing 10% FBS. Aliquots were stored at −80°C until use. Freshly thawed IL-2 was used for every experiment.

### In vitro culture

Purified T cells were labeled with CTV (Invitrogen) according to the manufacturer’s protocol. CTV-labeled cells were cultured in IMDM (Gibco) supplemented with 10% FBS (Gibco), 1x GlutaMAX (Gibco), 50 μM 2-mercaptoethanol (Sigma-Aldrich), 100 U/ml penicillin and streptomycin (Gibco), and 20 μg/ml gentamicin (Gibco) at 37°C/5% CO_2_, with or without IL-2. After 72 h, cells were collected for flow analysis. In some cases, OT-I cells were also stimulated with the ovalbumin epitope SIINFEKL (InvivoGen) at concentrations indicated within the figures.

### Flow cytometry

**Figure S4.**
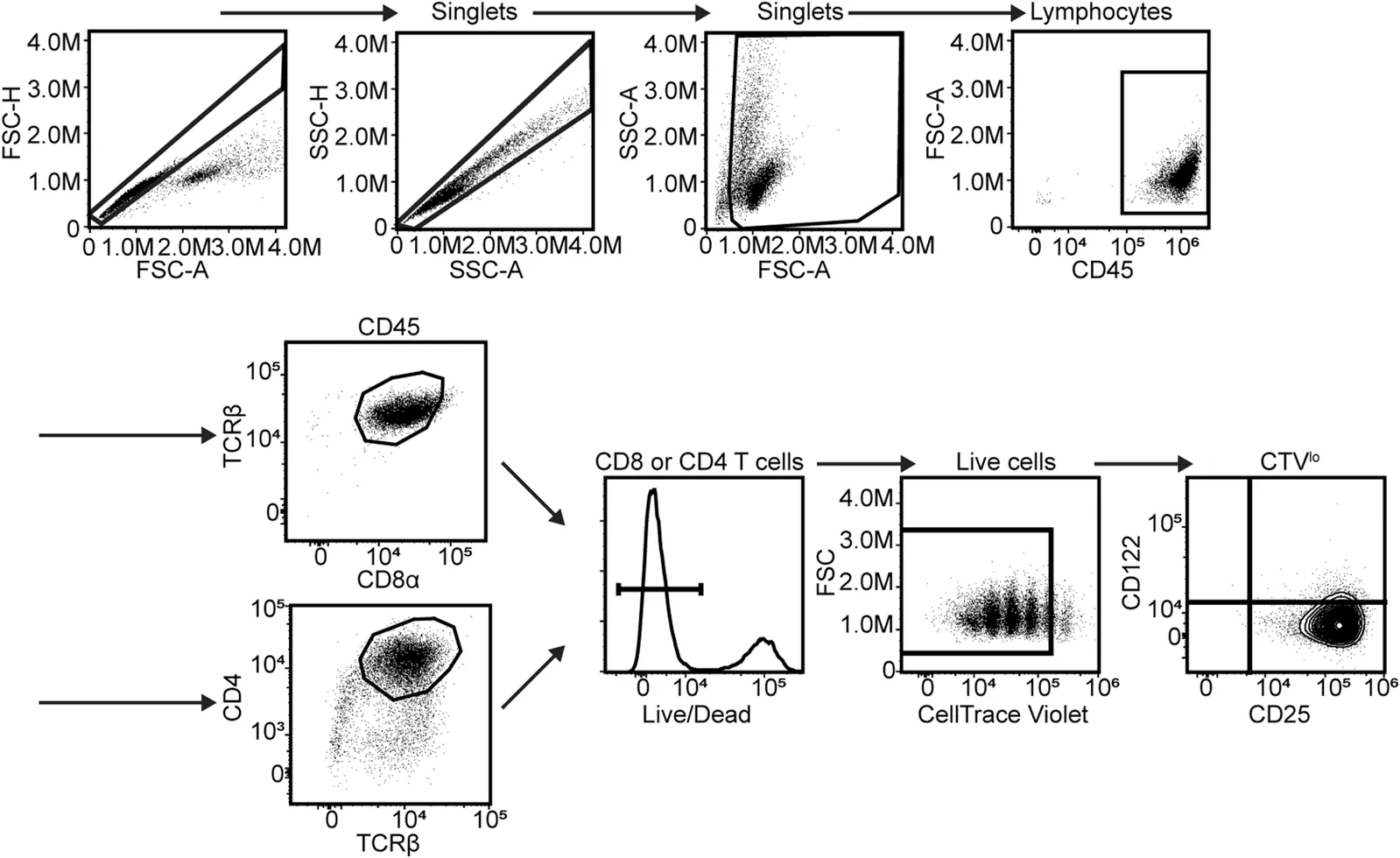
Generic gating strategy for flow cytometry after in vitro culture. A typical gating strategy that used post-culture conditions is shown.

The following fluorophore-conjugated antibodies were purchased from BioLegend or BD Biosciences: anti-CD45 (30-F11), CD8α (53-6.7), CD4 (RM4-5), CD25 (PC61), CD44 (IM7), CD122 (TM-β1), and TCRβ (H57-597). Live/Dead dye was purchased from Invitrogen. Briefly, cells were stained with antibodies at 4°C in the dark for 30 min. After washing, cells were fixed with 2% PFA (Electron Microscopy Science) at 4°C for 20 min. Data were collected on Aurora (Cytek) and analyzed with FlowJo software (Tree Star). A generic gating strategy is shown in Fig S4.

### Statistical analysis

All statistical analyses were performed in Prism (GraphPad Software). A *t* test was used for comparisons of two groups. One-way ANOVA with a post hoc *t* test was used for comparisons of more than two groups. **, *P* ≤ 0.01; ***, *P* ≤ 0.001; ****, *P* ≤ 0.0001.

## Supplementary Material

Reviewer comments

## Data Availability

The data that support the findings of this study and materials generated in this study are available on request from the corresponding author, BS Sheridan.
