# Supplementary material for: Murine IL-2 outperforms human IL-2 in supporting the survival of naïve mouse CD8 T cells in vitro
Source: Life Sci Alliance. 2026 Sep 24;9(12):e202603711. doi: 10.26508/lsa.202603711 (PMC13613269; doi:10.26508/lsa.202603711)
Supplement: Supplementary file 1 [file LSA-2026-03711_review_history.pdf]

Murine IL-2 outperforms human IL-2 in supporting the survival of naïve mouse CD8 T cells in vitro

Yangle Yu, Tete Obot, Madaleine Niznikiewicz, Craig Podszus, Julio Dos Santos, Elena Pobezinskaya, Priyadharshini Devarajan, Leonid Pobezinsky, and Brian Sheridan
DOI: https://doi.org/10.26508/lsa.202603711

Corresponding author(s): Brian Sheridan, Stony Brook University

Review Timeline:

Submission Date:	2026-03-23
Editorial Decision:	2026-06-01
Revision Received:	2026-07-31
Editorial Decision:	2026-09-03
Revision Received:	2026-09-08
Accepted:	2026-09-10

Scientific Editor: Tim Fessenden

Transaction Report:

(Note: With the exception of the correction of typographical or spelling errors that could be a source of ambiguity, letters and reports are not edited. The original formatting of letters and referee reports may not be reflected in this compilation.)

June 1, 2026

Re: Life Science Alliance manuscript #LSA-2026-03711

Prof. Brian S. Sheridan
Stony Brook University
Microbiology and Immunology
246 Centers for Molecular Medicine
Stony Brook, NY 11794-5120

Dear Dr. Sheridan,

Thank you for submitting your manuscript entitled "Murine IL-2 outperforms human IL-2 in supporting the survival of naïve mouse CD8 T cells in vitro". The manuscript has been evaluated by expert reviewers, whose reports are appended below. Reviewer 2 did not complete a report but privately conveyed that they were not in favor of publication. Unfortunately, after an assessment of the reviewer feedback, our editorial decision is against publication in Life Science Alliance at this time.

As you will see, both reviewers find the claims potentially important to the field but both find significant weaknesses in the evidence provided. They stress that a single source of IL-2 from each species was tested and note missing biological replicates (Reviewer 1) and missing details on cytokine production (Reviewer 3). These issues significantly reduce needed support for the main claim on cross-species stimulation by IL-2.

Although your manuscript is intriguing, I feel that the points raised by the reviewers are more substantial than can be addressed in a typical revision period. If you wish to expedite publication of the current data, it may be best to pursue publication at another journal.

Given our interest in the topic and its potential import, I would be open to re-submission to Life Science Alliance of a significantly revised and extended manuscript that fully addresses all reviewer concerns and is subject to further peer review. If you would like to resubmit this work to Life Science Alliance, you must first submit an appeal directly through our manuscript submission system. The appeal request must include a cover letter and a rebuttal with point-by-point responses to the reviewer comments. The appeal can optionally include a revised manuscript for editorial assessment, whereas a cover letter and response to reviewer comments are required.

Should you decide to pursue an appeal of this decision, it may be helpful to discuss the requested revisions in more detail via email or phone/videoconferencing. Please let me know which option you prefer, if any.

Regardless of how you choose to proceed, we hope that the comments below will prove constructive as your work progresses.

Thank you for thinking of Life Science Alliance as an appropriate venue to publish your work.

Sincerely,

Tim Fessenden
Scientific Editor
Life Science Alliance

Reviewer #1 (Comments to the Authors (Required)):

In their manuscript entitled "Murine IL-2 outperforms human IL-2 in supporting the survival of naïve mouse CD8 T cells in vitro", Yu, et al. compare the effects of murine and human IL-2 on mouse T cell survival, proliferation, and expression of IL-2 receptor subunits. The authors report that murine IL-2 is superior in enhancing the survival of primarily CD44lo CD8 mouse T cells, and that murine and human IL2 induce differential IL2-receptor subunit expression on these cells. I believe this work is of interest to the field, since many studies have used human IL2 to support in vitro cultures of mouse T cells without necessarily questioning whether the human IL2 is suboptimal for this purpose. Overall, the manuscript is clearly written and well organized, however I have some comments and concerns as outlined below.

- Fig. S1B: can the authors show a no IL2 control for CD44hi OT1? CD44hi cells may have some basal proliferation, and it would be nice to see what that looks like so the reader can adequately assess the contributions of human and mouse IL2 on the

proliferation of these cells.
- One issue I noted throughout the manuscript is that technical triplicates rather than biological replicates (ie: cells from different OT1 mice) are used and plotted in summary graphs. Without a sense of biological variation, it makes it difficult for the reader to critically evaluate some statements made in the manuscript. For example, IL2 primarily supports antigen-dependent T cell proliferation below 0.01ng/mL of the SIINFEKL peptide (Fig. 3A). Below this concentration range, it appears that human IL2 may somewhat enhance the proliferation, although the authors describe it as "comparable" to the effect of murine IL2. It becomes difficult for the reader to evaluate these statements when only an n=1 is shown. Were the authors to run an experiment with at least an n=3 (using OT1 T cells from 3 different mice), the reader would get a better sense of how comparable the proliferation is between human and mouse IL2 at these doses of SIINFEKL, and whether there is indeed no statistically significant difference. While I would be most assured by seeing biological replicates or independent experimental repeats in both Figures 1, 3 and S1, I think this is particularly necessary to support the statements made about Fig. 3A.
- Line 232-234: This line states that human and murine IL2 comparably upregulate CD25 in the presence of peptide. However, looking at the Fig. 3B, I would conclude that murine IL2 is better at inducing CD25 upregulation than human IL2 at lower doses of SIINFEKL as there is no CD25- population with murine IL2.
- I noticed the authors do not state the catalog number of the mouse and human IL2 from Peprotech. This is necessary so that the reader can look up how similar these cytokines are are in terms of the presence of carriers, endotoxin concentration etc. In addition, since this work can be of technical value to the community, I think the authors should compare murine and human IL2 from an additional commercial source, rather than just one manufacturer. Recombinant proteins from different sources can vary widely when it comes to their effect on cells in vitro, and the overall conclusion of this manuscript would be strengthened if a similar effect in Fig. 1 was seen with murine and human IL2 from an additional commercial source.

Reviewer #3 (Comments to the Authors (Required)):

The authors advocate that there is a meaningful difference in mouse and human IL-2 on mouse T cells. The data supporting this is not strong. There is not a substantial dose titration for key experiments. There is not a comparison of iL-2 from different vendors and with different production methodology. Rather, the differences are all from mouse and human IL-2 purchased from peprotech. The authors do not discuss how the IL-2 was produced and other manufacturing differences. Thus, are the differences observed due to the source of IL-2? Are the differences because a wider range of titrations was not performed?

Without addressing the above questions, this work would raise questions in the field based on very weak supporting data. In its current form, the author's conclusions may do more harm than benefit to the field.

Life Science Alliance

RE: LSA-2026-03711

Dear Editors,

I am writing to request an appeal of the decision to reject our manuscript titled "Murine IL-2 outperforms human IL-2 in supporting the survival of naïve mouse CD8 T cells *in vitro.*" In our view, the reviewer responses required minimal effort to address. We have now completed experiments to address all reviewers' concerns and have included our response attached to this cover letter. All our initial interpretations remain valid and were strengthened by inclusion of new data. We believe this manuscript will be of broad interest to the immunology community and serve as an important resource for many studies on T cell biology.

Addressing the Editor's Comments

We have improved the discussion about use of human IL-2 in murine in vitro CD8 T cell assays and focused on framing the problem for a broader audience. Concerning points raised by reviewer 2 associated with the unsurprising nature that murine and human IL-2 behave differently in culture, we completely agree. However, this does not change the fact that almost every paper that assesses murine CD8 T cells in vitro uses human IL-2 and no meaningful comparison has been undertaken in the literature in this way. We would argue that this manuscript would serve as a framework to determine whether scientists are performing the most meaningful experiments.

None of the material contained within this manuscript has been published or is under consideration elsewhere, including the Internet. Thank you for considering our manuscript for review. We appreciate your time and effort, and we look forward to hearing from you. I hope you find this manuscript of broad interest to *Life Science Alliance* readers and suitable for publication.

Thank you for considering this appeal. We are ready to submit our manuscript if our request is approved.

Sincerely,

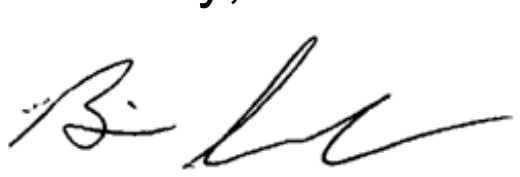

Brian S. Sheridan, Ph.D.
Associate Professor of Microbiology & Immunology

Center for Infectious Diseases
Stony Brook University
246 Centers for Molecular Medicine
Stony Brook, NY 11794-5120
Phone: 631-632-4459
Email: brian.sheridan@stonybrook.edu

Response to Reviewers' Concerns

Reviewer #1

In their manuscript entitled "Murine IL-2 outperforms human IL-2 in supporting the survival of naïve mouse CD8 T cells in vitro", Yu, et al. compare the effects of murine and human IL-2 on mouse T cell survival, proliferation, and expression of IL-2 receptor subunits. The authors report that murine IL-2 is superior in enhancing the survival of primarily CD44lo CD8 mouse T cells, and that murine and human IL2 induce differential IL2-receptor subunit expression on these cells. I believe this work is of interest to the field, since many studies have used human IL2 to support in vitro cultures of mouse T cells without necessarily questioning whether the human IL2 is suboptimal for this purpose. Overall, the manuscript is clearly written and well organized, however I have some comments and concerns as outlined below.

- Fig. S1B: can the authors show a no IL2 control for CD44hi OT1? CD44hi cells may have some basal proliferation, and it would be nice to see what that looks like so the reader can adequately assess the contributions of human and mouse IL2 on the proliferation of these cells.

We included the no-IL-2 control data and it's corresponding paired experimental groups for CD44^{hi} CD8 T cells as revised figure S1B.

- One issue I noted throughout the manuscript is that technical triplicates rather than biological replicates (ie: cells from different OT1 mice) are used and plotted in summary graphs. Without a sense of biological variation, it makes it difficult for the reader to critically evaluate some statements made in the manuscript. For example, IL2 primarily supports antigen-dependent T cell proliferation below 0.01ng/mL of the SIINFEKL peptide (Fig. 3A). Below this concentration range, it appears that human IL2 may somewhat enhance the proliferation, although the authors describe it as "comparable" to the effect of murine IL2. It becomes difficult for the reader to evaluate these statements when only an n=1 is shown. Were the authors to run an experiment with at least an n=3 (using OT1 T cells from 3 different mice), the reader would get a better sense of how comparable the proliferation is between human and mouse IL2 at these doses of SIINFEKL, and whether there is indeed no statistically significant difference. While I would be most assured by seeing biological replicates or independent experimental repeats in both Figures 1, 3 and S1, I think this is particularly necessary to support the statements made about Fig. 3A.

While the data included in the manuscript reflects technical replicates, all the experiments were independently repeated at least twice except for the P14 cells. We believe inclusion of P14 cells and endogenous CD8 T cells improves the rigor of the findings (essentially serving as additional biologic replicates to OT-I cells but with different specificities) but the Pi14 cells are not essential for the conclusions of the manuscript and have moved it to supplemental data (Fig. S2A). To specifically address the reviewer's concerns, we will include all independent experiments within the manuscript. Experiments repeated 2 times will be represented as 2 individual bar graphs, 1 for each experiment (Fig. 1C, S1, S2B, S2C). Experiments repeated more than 2 times will be pulled as cumulative bar graphs for statistical comparisons (Fig. 1A, 1B, 2C, 3C).

- Line 232-234: This line states that human and murine IL2 comparably upregulate CD25 in the presence of peptide. However, looking at the Fig. 3B, I would conclude that murine IL2 is better at inducing CD25 upregulation than human IL2 at lower doses of SIINFEKL as there is no CD25- population with murine IL2.

We revised the corresponding text to more accurately reflect the nuance of the experimental findings.

- I noticed the authors do not state the catalog number of the mouse and human IL2 from Peprotech. This is necessary so that the reader can look up how similar these cytokines are are in terms of the presence of carriers, endotoxin concentration etc. In addition, since this work can be of technical value to the community, I think the authors should compare murine and human IL2 from an additional commercial source, rather than just one manufacturer. Recombinant proteins from different sources can vary widely when it comes to their effect on cells in vitro, and the overall conclusion of this manuscript would be strengthened if a similar effect in Fig. 1 was seen with murine and human IL2 from an additional commercial source.

We have updated the methods section to include information on IL-2. For Peprotech, the catalog number for murine IL-2 is 212-12 and human IL-2 is 200-02. Both recombinant proteins are expressed by E. coli with ≥ 98% purity by SDS-PAGE gel and HPLC analyses. The endotoxin level is < 0.1 ng/µg of protein. Murine IL-2 is

reconstituted in water and then diluted in PBS containing 10% FBS. Human IL-2 is reconstituted in 100 mM Acetic Acid and then diluted in PBS containing 10% FBS following manufacturer's instruction.

We agree that reliance on single commercial source of IL-2 represented a limitation of the initial study. In the revised manuscript, we also evaluated murine and human IL-2 from STEMCELL (Cat# 78081 and 78220, respectively). Similar to PeproTech, both human and murine IL-2 from STEMCELL is expressed by E. coli. Purity is >95% and endotoxin levels are <0.1 ng/µg of protein. The observed results were consistent between vendors strengthening our interpretations.

<u>*Reviewer #3*</u>

The authors advocate that there is a meaningful difference in mouse and human IL-2 on mouse T cells. The data supporting this is not strong. There is not a substantial dose titration for key experiments. There is not a comparison of iL-2 from different vendors and with different production methodology. Rather, the differences are all from mouse and human IL-2 purchased from peprotech. The authors do not discuss how the IL-2 was produced and other manufacturing differences. Thus, are the differences observed due to the source of IL-2?

We have updated the methods section to include information on IL-2. For Peprotech, the catalog number for murine IL-2 is 212-12 and human IL-2 is 200-02. Both recombinant proteins are expressed by E. coli with ≥ 98% purity by SDS-PAGE gel and HPLC analyses. The endotoxin level is < 0.1 ng/µg of protein. Murine IL-2 is reconstituted in water and then diluted in PBS containing 10% FBS. Human IL-2 is reconstituted in 100 mM Acetic Acid and then diluted in PBS containing 10% FBS following manufacturer's instruction.

We agree that reliance on single commercial source of IL-2 represented a limitation of the initial study. In the revised manuscript, we also evaluated murine and human IL-2 from STEMCELL (Cat# 78081 and 78220, respectively). Similar to PeproTech, both human and murine IL-2 from STEMCELL is expressed by E. coli. Purity is >95% and endotoxin levels are <0.1 ng/µg of protein. The observed results were consistent between vendors strengthening our interpretations.

Are the differences because a wider range of titrations was not performed?

In Figure 2, we compared the effect of varying concentration of murine or human IL-2 in supporting naïve OT-I cells in vitro. 50-200 U/mL of IL-2 appears to be the most commonly used concentration of human IL-2 in the culture of CD8 T cells (Lee et al., 2014; Mandal et al., 2023; Yu et al., 2016). We observed a dose-dependent improvement in OT-I cell viability with increasing concentration of both cytokines. However, murine IL-2 achieved >80% viability at 150 U/mL, and increasing the concentration to 200 U/mL didn't produce an increase of viability of OT-I cells. In contrast, human IL-2 required substantially higher concentrations (800 U/mL) to achieve a comparable level of cell viability. These findings were valid for IL-2 from PeproTech and STEMCELL. This data suggests that affinity for the receptor is the primary driver of the differences observed in the response to IL-2.

July 24, 2026

MS: LSA-2026-03711

Prof. Brian S. Sheridan
Stony Brook University
Microbiology and Immunology
246 Centers for Molecular Medicine
Stony Brook, NY 11794-5120

Dear Dr. Sheridan,

Thank you for your letter appealing our prior decision on your manuscript entitled "Murine IL-2 outperforms human IL-2 in supporting the survival of naïve mouse CD8 T cells in vitro". Our decision has now been reconsidered in view of the revisions outlined in your rebuttal letter. I am pleased to let you know that have decided to invite a revised manuscript for re-review. Please note we will be looking for strong reviewer support of the revised work in order to proceed towards publication.

Please use the following link to submit your revised manuscript:
https://lsa.msubmit.net/cgi-bin/main.plex?el=A3Na2BkF3A1rTQ6I4B9ftdSr3nExFRb2caiP8PzIC06AZ

Yours sincerely,

Tim Fessenden
Scientific Editor
Life Science Alliance

Response to Reviewers' Concerns

Reviewer #1

In their manuscript entitled "Murine IL-2 outperforms human IL-2 in supporting the survival of naïve mouse CD8 T cells in vitro", Yu, et al. compare the effects of murine and human IL-2 on mouse T cell survival, proliferation, and expression of IL-2 receptor subunits. The authors report that murine IL-2 is superior in enhancing the survival of primarily CD44lo CD8 mouse T cells, and that murine and human IL2 induce differential IL2-receptor subunit expression on these cells. I believe this work is of interest to the field, since many studies have used human IL2 to support in vitro cultures of mouse T cells without necessarily questioning whether the human IL2 is suboptimal for this purpose. Overall, the manuscript is clearly written and well organized, however I have some comments and concerns as outlined below.

- Fig. S1B: can the authors show a no IL2 control for CD44hi OT1? CD44hi cells may have some basal proliferation, and it would be nice to see what that looks like so the reader can adequately assess the contributions of human and mouse IL2 on the proliferation of these cells.

We included the no IL-2 control data as revised figure S1B 'None'. CD44hi OT-I cells do not undergo basal proliferation in the absence of IL-2. Human IL-2 appears to induce slightly more proliferation than no IL-2 but was not significantly different when 4 independent experiments were pooled.

- One issue I noted throughout the manuscript is that technical triplicates rather than biological replicates (ie: cells from different OT1 mice) are used and plotted in summary graphs. Without a sense of biological variation, it makes it difficult for the reader to critically evaluate some statements made in the manuscript. For example, IL2 primarily supports antigen-dependent T cell proliferation below 0.01ng/mL of the SIINFEKL peptide (Fig. 3A). Below this concentration range, it appears that human IL2 may somewhat enhance the proliferation, although the authors describe it as "comparable" to the effect of murine IL2. It becomes difficult for the reader to evaluate these statements when only an n=1 is shown. Were the authors to run an experiment with at least an n=3 (using OT1 T cells from 3 different mice), the reader would get a better sense of how comparable the proliferation is between human and mouse IL2 at these doses of SIINFEKL, and whether there is indeed no statistically significant difference. While I would be most assured by seeing biological replicates or independent experimental repeats in both Figures 1, 3 and S1, I think this is particularly necessary to support the statements made about Fig. 3A.

We completely agree with the reviewer that presenting biological variation and independent experimental repeats is essential for rigorous evaluation. While the original figures displayed technical triplicates from individual representative runs, all experiments were independently repeated at least twice (and in many cases, three or more times).

To ensure full transparency and provide the necessary biological context, we have updated the manuscript to incorporate all independent experimental repeats as follows:

- *Cumulative Data (n≥3 independent experiments): For experiments repeated three or more times, we have pooled the data into cumulative graphs and performed statistical comparisons to rigorously evaluate differences (Figures 1A, 1B, 2, 3, and S1).*
- *Individual Biological Repeats (n=2 independent experiments): For panels representing experiments repeated twice, we now present both independent biological runs side-by-side to illustrate the consistency of the biological effect (Figures 1C, S2B, and S2C).*
- *Figure S3: Figure S3 reflects two independent experiments and represents a more comprehensive dose response curve than shown in Figure 3. Only 1 is shown since these data are incorporated into the cumulative data of Figure 3.*

Finally, to streamline the main narrative and keep the focus on robust biological replicates, the P14 cell data (which was performed once as an orthogonal validation model) has been moved to the supplemental data (Figure S2A). We believe these modifications provide a transparent view of the biological variation and firmly support our conclusions.

- Line 232-234: This line states that human and murine IL2 comparably upregulate CD25 in the presence of peptide. However, looking at the Fig. 3B, I would conclude that murine IL2 is better at inducing CD25 upregulation than human IL2 at lower doses of SIINFEKL as there is no CD25- population with murine IL2.

We revised the corresponding text to more accurately reflect the nuance of the experimental findings.

- I noticed the authors do not state the catalog number of the mouse and human IL2 from Peprotech. This is necessary so that the reader can look up how similar these cytokines are in terms of the presence of carriers, endotoxin concentration etc. In addition, since this work can be of technical value to the community, I think the authors should compare murine and human IL2 from an additional commercial source, rather than just one manufacturer. Recombinant proteins from different sources can vary widely when it comes to their effect on cells in vitro, and the overall conclusion of this manuscript would be strengthened if a similar effect in Fig. 1 was seen with murine and human IL2 from an additional commercial source.

We have updated the methods section to include information on IL-2. For Peprotech, the catalog number for murine IL-2 is 212-12 and human IL-2 is 200-02. Both recombinant proteins are expressed by E. coli with ≥ 98% purity by SDS-PAGE gel and HPLC analyses. The endotoxin level is < 0.1 ng/μg of protein. Murine IL-2 is reconstituted in water and then diluted in PBS containing 10% FBS. Human IL-2 is reconstituted in 100 mM Acetic Acid and then diluted in PBS containing 10% FBS following manufacturer's instruction.

We agree that reliance on single commercial source of IL-2 represented a limitation of the initial study. In the revised manuscript, we also evaluated murine and human IL-2 from STEMCELL (Cat# 78081 and 78220, respectively). Similar to PeproTech, both human and murine IL-2 from STEMCELL is expressed by E. coli. Purity is >95% and endotoxin levels are <0.1 ng/μg of protein. The observed results were consistent between vendors strengthening our interpretations.

<u>*Reviewer #3*</u>

The authors advocate that there is a meaningful difference in mouse and human IL-2 on mouse T cells. The data supporting this is not strong. There is not a substantial dose titration for key experiments. There is not a comparison of iL-2 from different vendors and with different production methodology. Rather, the differences are all from mouse and human IL-2 purchased from peprotech. The authors do not discuss how the IL-2 was produced and other manufacturing differences. Thus, are the differences observed due to the source of IL-2?

We have updated the methods section to include information on IL-2. For Peprotech, the catalog number for murine IL-2 is 212-12 and human IL-2 is 200-02. Both recombinant proteins are expressed by E. coli with ≥ 98% purity by SDS-PAGE gel and HPLC analyses. The endotoxin level is < 0.1 ng/μg of protein. Murine IL-2 is reconstituted in water and then diluted in PBS containing 10% FBS. Human IL-2 is reconstituted in 100 mM Acetic Acid and then diluted in PBS containing 10% FBS following manufacturer's instruction.

We agree that reliance on single commercial source of IL-2 represented a limitation of the initial study. In the revised manuscript, we also evaluated murine and human IL-2 from STEMCELL (Cat# 78081 and 78220, respectively). Similar to PeproTech, both human and murine IL-2 from STEMCELL is expressed by E. coli. Purity is >95% and endotoxin levels are <0.1 ng/μg of protein. The observed results were identical between vendors strengthening our interpretations and conclusions.

Are the differences because a wider range of titrations was not performed?

In Figure 2, we compared the effect of varying concentration of murine or human IL-2 in supporting naïve OT-I cells in vitro. While standard literature protocols typically utilize 50-200 U/mL of human IL-2 for murine CD8 T cell cultures, our evaluation extended significantly beyond this conventional range. Our findings demonstrated distinct functional profiles. Murine IL-2 reached a maximum viability plateau (~80%) at ~100 U/mL. Human IL-2 displayed a right-shifted curve, requiring an eight-fold higher concentration (800 U/mL) to achieve a comparable biological effect. Importantly, this necessity for higher human IL-2 concentrations was consistently observed using reagents from two independent manufacturers (PeproTech and STEMCELL Technologies), ruling out vendor-specific variation. Therefore, the differences described are not due to a restricted titration range but rather reflect a fundamental difference in functional potency on murine cells, likely driven by a lower affinity of human IL-2 for the murine IL-2 receptor complex.

Without addressing the above questions, this work would raise questions in the field based on very weak supporting data. In its current form, the author's conclusions may do more harm than benefit to the field.

We appreciate the reviewer's perspective regarding the scope and implications of our conclusions. The primary objective of this study is to address a deeply entrenched, historical convention within the T cell community. The widespread use of human IL-2 in murine T cell cultures is frequently driven by historical precedent and cost

efficiencies rather than optimized biological assessment. Our data aims to provide a practical resource that encourages laboratories to re-evaluate their IL-2 dosing strategies or consider transitioning to murine IL-2. Regarding the choice of cytokine vendor, we focused on PeproTech reagents because they represent the predominant standard across a vast majority of T cell biology laboratories; thus, focusing on this manufacturer ensures maximum immediate relevance to the field. That being said, we take the reviewer's concerns regarding data strength seriously. To ensure our conclusions are robust and properly contextualized, we have assessed all experiments with another commercial vendor as indicated above. We believe these revisions clarify the scope of our study and prevent any potential misinterpretation.

September 3, 2026

RE: Life Science Alliance Manuscript #LSA-2026-03711R-A

Prof. Brian S. Sheridan
Stony Brook University
Microbiology and Immunology
246 Centers for Molecular Medicine
Stony Brook, NY 11794-5120

Dear Dr. Sheridan,

Thank you for submitting your revised manuscript entitled "Murine IL-2 outperforms human IL-2 in supporting the survival of naïve mouse CD8 T cells in vitro". Your manuscript was returned to the original Reviewers 1 and 3, whose comments are below. As you will see, the reviewers diverged in their appraisal of the revised work. We appreciate the close attention to detail by Reviewer 3 related to T cell isolation and the cell culture timecourse, while noting that their prior concerns on IL-2 titration and the sources of recombinant IL-2 have been addressed. We concur with Reviewer 1 that the claims related to CD4 T cells must be adjusted in light of the data shown, and that the papers noted by Reviewer 3 must be discussed. Finally, this work is primarily motivated by a widespread use of human IL-2 that is not adequately documented. Please cite literature to support the claim on lines 70-71.

In view of the support of Reviewer 1 and revisions in place requested by Reviewer 3, we will be glad to publish your paper in Life Science Alliance pending the above revisions as well as final changes necessary to meet our formatting guidelines.

MANUSCRIPT ORGANIZATION AND FORMATTING:

To avoid unnecessary delays in the acceptance and publication of your paper, please read the following information carefully. Full guidelines are available on our Instructions for Authors page, https://www.life-science-alliance.org/authors

Submission of a paper that does not conform to Life Science Alliance guidelines will delay the acceptance of your manuscript.

-Please upload your main manuscript text as an editable doc file.
-Please add the X and Bluesky handles of your host institute/organization, as well as your own and/or one of the authors, in our system.
-Please remove the eTOC summary from the manuscript file.
-Please use the [10 author names et al.] format in your references (i.e., limit the author names to the first 10).
-There is a call-out for figure S5, and the call-out for figure S4 is missing. Please correct.

We encourage our authors to provide original source data, particularly uncropped/-processed electrophoretic blots and spreadsheets for the main figures of the manuscript. If you would like to add source data, we would welcome one PDF/Excel-file per figure for this information. These files will be linked as supplementary "Source Data" files.

It is Life Science Alliance policy that if requested, original data images must be made available to the editors. Failure to provide original images upon request will result in unavoidable delays in publication. Please ensure that you have access to all original data images prior to final submission.

If you are planning a press release on your work, please inform us immediately to allow informing our production team and scheduling a release date.

We welcome submissions of potential cover images for the issue of LSA in which your work would appear. If you have high quality images associated with this work, please feel free to email these, with a caption, to the journal office.

LSA encourages authors to provide a 30-60 second video where the study is briefly explained. These videos will be appear embedded with the manuscript online at Life Science Alliance, and on social media to promote the published paper and authors (for examples, see https://docs.google.com/document/d/1-UWCfbE4pGcDdcgzcmiuJl2XMBJnxKYeqRvLLrLSo8s/edit?usp=sharing). Corresponding or first-authors are welcome to submit the video. Please submit only one video per manuscript. The video can be emailed to contact@life-science-alliance.org

FINAL FILES:

To upload the final version of your manuscript, please log in to your account: https://lsa.msubmit.net/cgi-bin/main.plex
You will be guided to complete the submission of your revised manuscript and to fill in all necessary information. Please get in touch in case you do not know or remember your login name.

The following items are required for acceptance.

-- An editable version of the final text (.DOC or .DOCX) is needed for copyediting (no PDFs).

-- High-resolution figure, supplementary figure and video files uploaded as individual files: See our detailed guidelines for preparing your production-ready images, https://www.life-science-alliance.org/authors

-- Summary blurb (enter in submission system): A short text summarizing in a single sentence the study (max. 200 characters including spaces). This text is used in conjunction with the titles of papers, hence should be informative and complementary to the title. It should describe the context and significance of the findings for a general readership; it should be written in the present tense and refer to the work in the third person. Author names should not be mentioned.

The license to publish form must be signed before your manuscript can be sent to production. A link to the license to publish form will be available to the corresponding author only. Please take a moment to check your funder requirements.

Reviews, decision letters, and point-by-point responses associated with peer-review at Life Science Alliance will be published, alongside the manuscript. If you do want to opt out of having the reviewer reports and your point-by-point responses displayed, please let us know immediately.

Thank you for your attention to these final processing requirements. Please revise and format the manuscript and upload materials as soon as you are able.

Thank you for this interesting contribution to the literature. We look forward to publishing your paper in Life Science Alliance.

Sincerely,

Tim Fessenden
Scientific Editor
Life Science Alliance

Reviewer #1 (Comments to the Authors (Required)):

Overall, the revised manuscript by Yu et al., on the impact of murine vs human IL2 on T cell survival sufficiently addressed my previous concerns, namely showing biological replicates and that the differential effect of murine and human IL2 on T cell survival is maintained across different cytokine manufacturers.

Prior to publication, I believe the authors should revise the conclusions regarding the differential effect of murine and human IL2 on CD4 T cell survival (lines 128 - 133). While the authors claim that murine IL2 provides a greater protective effect than human IL2 in HNT cells, experiment 2 in Fig. S2B does not show a statistically significant difference (at least as far as the reader can tell from the plot). If the result only held in one of two independent experiments shown then I think the authors should state that there is not a clear effect on CD4 T survival and possibly revise the last statement in that paragraph to: Altogether, these results suggest that murine IL-2 most effectively supports the survival of CD44lo CD8+ T cells in vitro.

I otherwise have no objection to publication of this manuscript.

Reviewer #3 (Comments to the Authors (Required)):

The authors propose a key conclusion that mouse IL-2 is more effective than human IL-2 at inducing survival of mouse naïve CD8+ T cells. In response to the prior review, the authors have performed experiments with an additional source of IL-2.

Unfortunately, the major concerns with the manuscript remain unresolved. The authors make broad conclusions regarding differences between mouse and human IL-2 that are not sufficiently supported by the experimental data or placed in the context of the existing literature.

The experiments assess the viability of mouse CD8+ T cells isolated by MACS bead purification. Even under optimal conditions,

this approach generally yields approximately 90% purity, yet the authors do not provide data documenting the purity of the isolated population. Because survival is assessed only after three days of culture, substantial changes may have occurred during this period, including the production of secondary cytokines that could contribute to cell survival. A time-course experiment would therefore be important to determine when the differential effects of mouse and human IL-2 emerge. In addition, the IL-2 titration covers a relatively narrow concentration range, and comparable titrations using multiple independent sources of mouse and human IL-2 are not presented.

There is a lot of literature comparing human and mouse IL-2 in different assays, and these papers are not discussed. For example PMID 3873588 and 3493289. The latter paper is cited, but there is not a discussion of why mouse iL-2 and human IL-2 induce a similar effect and different than what is found in the current paper.

More broadly, the central issue is that the authors make the general claim that mouse IL-2 is more effective than human IL-2 at promoting the survival of naïve CD8+ T cells from mice. While this could be true, it cannot be established from the data currently presented.

Reviewer 1
Prior to publication, I believe the authors should revise the conclusions regarding the differential effect of murine and human IL2 on CD4 T cell survival (lines 128 - 133). While the authors claim that murine IL2 provides a greater protective effect than human IL2 in HNT cells, experiment 2 in Fig. S2B does not show a statistically significant difference (at least as far as the reader can tell from the plot). If the result only held in one of two independent experiments shown then I think the authors should state that there is not a clear effect on CD4 T survival and possibly revise the last statement in that paragraph to: Altogether, these results suggest that murine IL-2 most effectively supports the survival of CD44lo CD8+ T cells in vitro.

We thank the reviewer for this helpful observation and agree with their assessment. We have revised the text to reflect that murine IL-2 does not provide a consistent survival advantage over human IL-2 in CD4 T cells.

Reviewer 3
The experiments assess the viability of mouse CD8+ T cells isolated by MACS bead purification. Even under optimal conditions, this approach generally yields approximately 90% purity, yet the authors do not provide data documenting the purity of the isolated population.

We appreciate the reviewer raising this point regarding population purity. As noted in our Materials and Methods section, our MACS bead isolation routinely yields >95% purity. Importantly, to eliminate potential confounders, every experiment utilizes a split-sample design in which cells isolated from a single MACS purification are divided equally into the murine and human IL-2 treatment conditions. Because both treatment groups originate from the exact same cell pool, any minor non-target populations are identical across conditions. Furthermore, MACS isolation represents a standard methodology utilized by the vast majority of T cell laboratories. Conducting our experiments using this standard approach ensures that our findings serve as a directly applicable resource for the field.

Because survival is assessed only after three days of culture, substantial changes may have occurred during this period, including the production of secondary cytokines that could contribute to cell survival.

We appreciate the reviewer's point regarding potential downstream or secondary cell-culture dynamics during a 3-day incubation period. While secondary autocrine or paracrine factors could contribute to overall persistence over this timeframe, we emphasize that the cytokine source (murine vs. human IL-2) is the single variable manipulated between culture conditions. Consequently, any downstream secondary cytokines or kinetic dynamics that emerge during culture are downstream biological consequences initiated by the initial IL-2 source. The primary objective of this work is to provide a practical benchmark for the T cell community regarding net functional outcomes under standard culture conditions. Whether the superior survival provided by murine IL-2 is mediated strictly through direct intracellular signaling or involves downstream culture dynamics, the practical conclusion that murine IL-2 is functionally superior for maintaining mouse CD8 T cell viability in vitro *remains identical.*

A time-course experiment would therefore be important to determine when the differential effects of mouse and human IL-2 emerge.

We thank the reviewer for this suggestion regarding time-course dynamics. While tracking early kinetic divergence would provide further temporal details, the net functional outcome, that murine IL-2 delivers superior viability for mouse CD8 T cells over the culture period, would remain unchanged.

In addition, the IL-2 titration covers a relatively narrow concentration range, and comparable titrations using multiple independent sources of mouse and human IL-2 are not presented.

Our titration spanned the complete functional window for both cytokines, from baseline (~10% viability) to maximal saturation (~80% viability). Murine IL-2 reached saturation at 200 U/mL, whereas human IL-2 required extension up to 800 U/mL to reach an equivalent level. Figure 2C presents data from four independent experiments utilizing recombinant cytokines from two separate commercial sources. Because these titrations capture maximum response plateaus across independent vendors, we believe the data firmly support our conclusions regarding the lower functional potency of human IL-2 on murine CD8 T cells.

There is a lot of literature comparing human and mouse IL-2 in different assays, and these papers are not discussed. For example PMID 3873588 and 3493289. The latter paper is cited, but there is not a discussion of why mouse iL-2 and human IL-2 induce a similar effect and different than what is found in the current paper.

We appreciate the reviewer pointing out these relevant studies. We have updated the Discussion section to more meaningfully address PMID 3873588 and PMID 3493289. In these historical studies both human and murine IL-2 induce comparable proliferative responses, a result that aligns with our own stimulation data. In contrast, our study focuses on the survival of unstimulated CD8 T cells and the phenotype of CD25 and CD122 expression after stimulation which was not assessed in these papers.

More broadly, the central issue is that the authors make the general claim that mouse IL-2 is more effective than human IL-2 at promoting the survival of naïve CD8+ T cells from mice. While this could be true, it cannot be established from the data currently presented.

For the above indicated reasons, we respectfully disagree with the reviewer's assertion that our data cannot establish that murine IL-2 is more effective than human IL-2 at promoting the survival of naïve mouse CD8 T cells.

September 10, 2026

RE: Life Science Alliance Manuscript #LSA-2026-03711RR

Prof. Brian S. Sheridan
Stony Brook University
Microbiology and Immunology
246 Centers for Molecular Medicine
Stony Brook, NY 11794-5120

Dear Dr. Sheridan,

Thank you for submitting your Research Article entitled "Murine IL-2 outperforms human IL-2 in supporting the survival of naïve mouse CD8 T cells in vitro". It is a pleasure to let you know that your manuscript is now accepted for publication in Life Science Alliance. Congratulations on this interesting work.

The final published version of your manuscript will be deposited by us to PubMed Central upon publication.

Your manuscript will now progress through copyediting and proofing. It is journal policy that authors provide original data upon request.

Your article will publish open access upon publication under a CC-BY license.

Reviews, decision letters, and point-by-point responses associated with peer-review at Life Science Alliance will be published, alongside the manuscript. As noted in our prior correspondence, this includes responses to the most recent reviewer comments on your revised manuscript.

IMPORTANT: If you will be unreachable at any time, please provide us with the email address of an alternate author. Failure to respond to routine queries may lead to unavoidable delays in publication.

Scheduling details will be available from our production department. You will receive proofs shortly before the publication date. Only essential corrections can be made at the proof stage so if there are any minor final changes you wish to make to the manuscript, please let the journal office know now.

DISTRIBUTION OF MATERIALS:
Authors are required to distribute freely any materials used in experiments published in Life Science Alliance. Authors are encouraged to deposit materials used in their studies to the appropriate repositories for distribution to researchers.

You can contact the journal office with any questions, contact@life-science-alliance.org

Again, congratulations on a very nice paper. I hope you found the review process to be constructive and are pleased with how the manuscript was handled editorially. We look forward to future exciting submissions from your lab.

Sincerely,

Tim Fessenden
Scientific Editor
Life Science Alliance
